# Physiological Response of Cape Gooseberry Plants to *Fusarium oxysporum* f. sp. *physali*, Fusaric Acid, and Water Deficit in a Hydrophonic System

**DOI:** 10.3389/fpls.2021.702842

**Published:** 2021-08-05

**Authors:** Luis Alberto Mendoza-Vargas, Wendy Paola Villamarín-Romero, Anderson Steven Cotrino-Tierradentro, Joaquín Guillermo Ramírez-Gil, Cristhian Camilo Chávez-Arias, Hermann Restrepo-Díaz, Sandra Gómez-Caro

**Affiliations:** Universidad Nacional de Colombia, Sede Bogotá, Facultad de Ciencias Agrarias, Departamento de Agronomía, Bogotá, Colombia

**Keywords:** abiotic stress, biotic stress, growth and development, mycotoxin, vascular wilt

## Abstract

Cape gooseberry production has been limited by vascular wilt caused by *Fusarium oxysporum* f. sp. *physali* (Foph). Fusaric acid (FA) is a mycotoxin produced by many *Fusarium* species such as *F. oxysporum formae speciales*. The effects of the interaction between this mycotoxin and plants (such as cape gooseberry) under biotic stress (water deficit, WD) have been little explored. Three experiments were carried out. The objectives of this study were to evaluate (i) different Foph inoculum densities (1 × 10^4^ and 1 × 10^6^ conidia ml^−1^; experiment (1); (ii) the effect of times of exposure (0, 6, 9, and 12 h) and FA concentrations (0, 12.5, 25, 50, and 100 mg L^−1^; experiment (2), and (iii) the interaction between Foph (1 × 10^4^ conidia mL^−1^) or FA (25 mg L^−1^ × 9 h), and WD conditions (experiment 3) on the physiological (plant growth, leaf stomatal conductance (*g*_*s*_), and photochemical efficiency of PSII (F_v_/F_m_ ratio) and biochemical [malondialdehyde (MDA) and proline] responses of cape gooseberry seedling ecotype Colombia. The first experiment showed that Foph inoculum density of 1 × 10^6^ conidia ml^−1^ caused the highest incidence of the disease (100%). In the second experiment, *g*_*s*_ (~40.6 mmol m^−2^ s^−1^) and F_v_/F_m_ ratio (~0.59) decreased, whereas MDA (~9.8 μmol g^−1^ FW) increased in plants with exposure times of 9 and 12 h and an FA concentration of 100 mg L^−1^ compared with plants without FA exposure or concentrations (169.8 mmol m^−2^ s^−1^, 0.8, and 7.2 μmol g^−1^ FW for *g*_*s*_, F_v_/F_m_ ratio and MDA, respectively). In the last experiment, the interaction between Foph or FA and WD promoted a higher area under the disease progress curve (AUDPC) (Foph × WD = 44.5 and FA × WD = 37) and lower *g*_*s*_ (Foph × WD = 6.2 mmol m^−2^ s^−1^ and FA × WD = 9.5 mmol m^−2^ s^−1^) compared with plants without any interaction. This research could be considered as a new approach for the rapid scanning of responses to the effects of FA, Foph, and WD stress not only on cape gooseberry plants but also on other species from the Solanaceae family.

## Introduction

Cape gooseberry (*Physalis peruviana*) is a species from the South American Andes. This plant is mainly cultivated in the highlands of the Andean region and other subtropical areas around the world (Fischer et al., 2014; Thomas and Sepúlveda, 2014). This crop represents an important alternative in the economy of countries such as Colombia, Kenya, Zimbabwe, Australia, New Zealand, and Ecuador, since its fruits can be exported as a fresh product because of their nutritional characteristics (Fischer et al., 2014). Additionally, *P. peruviana* can be considered a source of bioactive compounds with a positive role in human health and applications in the medical industry (Thomas and Sepúlveda, 2014). In Colombia, this species faces an important phytosanitary problem caused by the fungus *Fusarium oxysporum* f. sp. *physali* (Foph) (vascular wilt) (Thomas and Sepúlveda, 2014; Simbaqueba et al., 2018). Vascular wilt has caused crop losses of up to 50% in affected production areas and migration to new sites since infective propagules can remain in the soil for long periods (Gordon and Martyn, 1997; Fischer et al., 2014, 2021; Husaini et al., 2018). Therefore, vascular wilt is the main constraint for cape gooseberry crop sustainability (Fischer and Miranda, 2012; Forero, 2014).

*Fusarium oxysporum* (Fo) is a widely distributed and important soil pathogen that can affect large numbers of plant species (Gordon and Martyn, 1997; Dean et al., 2012). This microorganism affects the growth, development, physiology, and biochemistry of its hosts. Fo generates the obstruction and damage of the vascular system; this affectation initially reduces water uptake and nutrient translocation and then causes wilting, chlorosis, and death in plants (Gordon and Martyn, 1997; Dean et al., 2012; Husaini et al., 2018). According to the plant host, different *formae speciales* of this pathogen have been reported (Kant et al., 2011). Foph was reported in 2018 in cape gooseberry (Simbaqueba et al., 2018, 2021). Foph causes disease symptoms such as chlorosis of lower leaves, plant wilting, leaf senescence, and, finally, plant death. These symptoms may appear only on one side of the plant (Forero, 2014; Giraldo-Betancourt et al., 2020; Fischer et al., 2021). In Colombia, the disease is currently present in most production areas where main crop regions (Department of Cundinamarca) show wilt incidence of over 50% (Forero, 2014).

Fungi of the genus *Fusarium* produce several mycotoxins that may promote vascular wilt in plant hosts (Berestetskiy, 2008; Wu et al., 2008a). Additionally, these mycotoxins can contribute to pathogenesis or virulence and competition with other organisms (Bell et al., 2003). Beauvericin, bikaverin, enniatins, fusarin C, isoverrucarol, moniliformin, naphthoquinone pigments, sambutoxin, wortmannin, fusaric acid, and fumonisins are some of the mycotoxins reported in Fo (Jennings, 2007; Srinivas et al., 2019; Zuriegat et al., 2021). Fusaric acid (FA, 5-n-butyl-2-pyridine carboxylic acid) is involved in the progression of vascular wilt in various crops, such as bananas (Liu et al., 2020; Portal-González et al., 2021) watermelon (Wu et al., 2008a), cucumber (Wang et al., 2014, 2015), tomatoes (Singh et al., 2017), faba beans (Li et al., 2021), and chickpea (Maharshi et al., 2021). FA induces programmed cell death, increases ROS production and lipid peroxidation (Jiao et al., 2014; Singh and Upadhyay, 2014), alters electrolyte leakage, and reduces photosynthetic pigments (Wu et al., 2008a,b; Singh et al., 2017). FA is considered a virulence factor, since low levels of this mycotoxin are associated with a drop in vascular wilt severity (Ding et al., 2018; López-Díaz et al., 2018; Gurdaswani et al., 2020; Liu et al., 2020; Shao et al., 2020). Some of the symptoms caused by FA include leaf turgor and chlorophyll loss, vascular tissue browning, and necrosis (Wu et al., 2008b; Singh et al., 2017). More pathogenic Fo strains may produce higher FA concentrations than those reported in studies under laboratory conditions (which range between 90 and 320 μg g^−1^ FA) (Bacon et al., 1996; Venter and Steyn, 1998). However, little is known about the role of FA in the Fo—*P. peruviana* interaction and the development of vascular wilt. Nevertheless, a study conducted by Izquierdo-García and Moreno-Velandia in 2018 (personal communication) confirmed the *in vitro* production of FA by Foph strain MAP5 in potato broth dextrose (PBD) media.

The understanding of the cape gooseberry-Foph interaction and its effects on plant physiological behavior have been studied during the last years (Enciso-Rodríguez et al., 2013; Simbaqueba et al., 2018; Chaves-Gómez et al., 2019; Cháves-Gómez et al., 2020). In the last decade, research about the interaction between water deficit and fusarium wilt incidence has also gained importance, since crop plants (such as cotton, tomato, and banana) have shown more severe disease development under water stress conditions (Ghaemi et al., 2011; Meddich et al., 2018; Orr and Nelson, 2018). Recent studies have been focused mainly on the effect of waterlogging and its impact on Foph incidence in cape gooseberry plants (Villarreal-Navarrete et al., 2017; Chávez-Arias et al., 2019, 2020). Those reports concluded that waterlogging caused a higher vascular wilt incidence, and that the physiological (stomatal conductance, F_v_/F_m_ ratio) and biochemical (MDA, and proline content) variables can be useful to evaluate the effect of single or combined stresses on this plant species. Environmental conditions such as temperature, water activity, and CO_2_ can modulate the production of *Fusarium* mycotoxins (fusaric and fusarinolic acids) (Fumero et al., 2020; Ladi et al., 2020). Also, water and salinity stress conditions affect Fo virulence, increasing FA production, and disease severity (Nurcahyani et al., 2019; Maharshi et al., 2021).

Climate change has generated more frequent drought events, affecting yields in multiple crop systems in Colombia (Ramírez-Gil and Morales-Osorio, 2018). Water deficit reduces plant growth, leaf expansion, biomass accumulation, chlorophyll content, and leaf gas exchange parameters (photosynthesis, stomatal conductance, and plant transpiration), and increases leaf temperature and trichome density in cape gooseberry plants (Segura-Monroy et al., 2015; Fischer and Melgarejo, 2020). However, information remains scarce regarding the role of FA as a virulence factor and its interaction with vascular wilt under water deficit conditions in cape gooseberry plants. FA has been used mainly in studies conducted to select materials with resistance to Fo under *in vitro* evaluations in carnation, celery, orchid, tomato, etc. (Svabova and Lebeda, 2005; Kant et al., 2011; Nurcahyani et al., 2019). Studies on FA may also be carried out to understand the influence of toxin-related damage or toxin-producing fungi on plant disease development.

It is necessary to develop methodologies that allow the rapid evaluation of infection processes by Fo in cape gooseberry plants. The evaluation of the effects of FA as a virulence factor to simulate pathogen infections may contribute to elucidate cape gooseberry responses to Fo–host interactions under water stress conditions. This way, FA can be used as a plant biomarker in studies on abiotic and biotic stress interactions in plants (Suharyanto et al., 2015). For this reason, the objectives of this study were to evaluate (i) different Foph inoculum densities (experiment 1); (ii) the effect of times of exposure and FA concentrations (experiment 2), and (iii) the interaction between Foph or FA (with the latter as a plant biomarker) and WD stress conditions (experiment 3) on the physiological (plant growth, gas exchange parameters, and photochemical efficiency of PSII) and biochemical (MDA and proline) responses of cape gooseberry seedling ecotype Colombia.

## Materials and Methods

### General Growth Conditions and Pathogen Inoculation

For all the experiments, a hydroponic system in 2.8-L glass containers was utilized, using a nutrient solution based on liquid 40N-4P-20K fertilizer containing micronutrients (NutriPonic, Walco S.A., Bogotá D.C., Colombia) at a rate of 2.5 ml L^−1^. The pH of the solution ranged from 5.5 to 6, and its concentration was as follows: 2.08 mM Ca (NO_3_)_2_·_4_H_2_O, 1.99 mM MgSO_4_·7H_2_O, 2 mM NH_4_H_2_PO_4_, 10.09 mM KNO_3_, 46.26 nM H_3_BO_3_, 0.45 nM Na_2_MoO_4_·2H_2_O, 0.32 nM CuSO_4_·5H2O, 9.19 nM MnCl_2_·4H_2_O, 0.76 nM ZnSO_4_·7H_2_O, and 19.75 nM FeSO_4_·H_2_O. The nutrient solution was refilled every 3 days to maintain a constant volume (2.5 L) in each container and was always aerated by an electric air pump.

We used cape gooseberry ecotype Colombia seedlings with three to four fully expanded leaves and purchased at a local nursery. The plants were arranged in the system using an expanded polystyrene sheet. Five plants were arranged in each container using an oxygen diffuser with a capacity of 7.8 L min^−1^ (Active Aqua Air Pump, Hydrofarm, Petaluma, CA, United States). The system was established under growth room conditions, with an average temperature of 22°C ± 0.8, 75% ± 5 relative humidity, and a 12 h artificial photoperiod using incandescent lamps supplying 800 μmol m^−2^ s^−1^ photosynthetically active radiation (PAR). The plants were kept in the containers with distilled water for 4 days before starting the treatments in each of the experiments for plants to adapt to the experimental conditions.

*Fusarium oxysporum* f. sp. *physali* (Foph) strain MAP5 (Laboratory of Biological Control, Agrosavia) was used as the pathogen source. The strain underwent previous morphological, molecular, and pathogenic characterization on cape gooseberry plants (Simbaqueba et al., 2018; Chaves-Gómez et al., 2019; Chávez-Arias et al., 2020). The pathogen was multiplied in malt extract (ME) liquid medium (Oxoid®, Thermo Fisher Scientific, Waltham, MA, United States) with constant stirring at 125 rpm, under darkness at 25°C for 10 days. Vascular damage in cape gooseberry plants was determined by the observation of cross-sections of the root-stem transition zone of each treatment. The presence of Foph in inoculated and non-inoculated plants was confirmed by isolating the pathogen in potato dextrose agar (PDA) (Oxoid®, Thermo Fisher Scientific, Waltham, MA, United States) medium from roots, tissue from the basal stem zone, and the root-stem transition region, previously subjected to a disinfection process (Narayanasamy, 2001). Petri dishes were incubated at 25°C. In all the experiments, a cape gooseberry plant was considered the experimental unit.

### Experiment 1. Evaluation of Cape Gooseberry Responses to *F. oxysporum* f. sp. *physali* Inoculum Densities

The plants were exposed to a permanent inoculum of Foph in the hydroponic system solution. This procedure was carried out by adding microconidia of the pathogen to each of the containers. Microconidia were obtained by the procedure of Foph multiplication in ME media (previously explained). Two inoculum densities, 1 × 10^4^ and 1 × 10^6^ conidia ml^−1^, were evaluated according to results obtained in previous studies (Urrea et al., 2011; Ding et al., 2014; Mayorga-Cubillos et al., 2019). These two Foph inoculum densities were considered to determine the more suitable to be used in the following experiments. The inoculum concentration for each treatment was adjusted by hemocytometer counting (Neubauer, VWR, Darmstadt, Germany). The response variables were evaluated at 0, 3, 7, 10, and 13 days after treatment (DAT), considering one cape gooseberry plant as the experimental unit, with three replicates per treatment and non-inoculated plants as control. Three replicates were used per treatment, and the experiment was conducted with a completely randomized design (CRD).

### Experiment 2. Evaluation of Exposure Times and Fusaric Acid Concentrations

Four FA (Sigma-Aldrich®, St. Louis, MI, United States; Merck, Kenilworth, NJ, United States) concentrations (12.5, 25, 50, and 100 mg L^−1^) and three exposure times (6, 9, and 12 h) were evaluated according to the reports of Wang et al. (2013) and Wang et al. (2014) to estimate the physiological behavior of cape gooseberry plants to the toxin. The FA solutions were prepared in 500 ml of sterile distilled water and stirred for 10 min. Right after the adaptation period, the plants were exposed to FA by root dipping in a glass beaker (500 ml) for the time required according to the treatments. Then, the FA-treated plants were transferred to containers with the nutrient solution until the end of the experiment (9 DAT). Plants without exposure to FA were only dipped in sterile distilled water and used as control. The experiment was established randomly in a 3 × 2 complete factorial arrangement with three replicates and one cape gooseberry plant as an experimental unit. The response variables were assessed at 0, 3, 6, and 9 DAT, except for the biochemical response variables, which were measured at the end of the experiment (9 DAT).

### Experiment 3. Conditions of Water Deficit and Exposure to Fusaric Acid and Fusarium

The plants were initially preconditioned with 5% polyethylene glycol (PEG 6000®, PanReac, Barcelona, Spain) to simulate water deficit (WD) by the addition of the compound to the nutrient solution in the containers for 6 days. The PEG concentration was selected, as it simulates a water deficit condition in plants (DeLaat et al., 2014; Sánchez-Reinoso et al., 2018). Subsequently, the nutrient solution with PEG was replaced with the nutrient solution without PEG in the containers, where the plants remained until the end of the trial (12 DAT). According to the results from experiments 1 and 2, pathogen inoculation was performed using permanent inoculum in the hydroponic system (1 × 10^4^ conidia ml^−1^), and plant roots were exposed to 25 mg L^−1^ of FA for 9 h. The established treatments were as follows: (i) plants with WD, (ii) plants with FA, (iii) plants with Foph, (iv) plants with combined stress (FA + WD); (v) plants with combined stress (Foph + WD), and (vi) plants without exposure to FA, Foph, and WD as control. In the FA + WD treatment, root dipping in FA and plant set was carried out as mentioned in experiment 2. Then, the FA-treated plants were placed in the containers with nutrient solution + PEG (WD). For the Foph + WD treatment, the inoculation of the pathogen was carried out 3 days before the start of the WD condition. Day zero of the experiment was considered as the time when the exposure of the plants to the water stress condition started. The experiment concluded 15 DAT when the Foph + WD plants displayed wilting symptoms or death. The response variables were evaluated at 0, 3, 6, 9, 12, and 15 DAT except for biochemical response, which was measured at the end of the experiment (15 DAT). Three replicates were used per treatment, and the experiment was established in a completely randomized design with a 2 × 2 factorial arrangement.

### Determination of Area Under the Disease Progress Curve and Vascular Browning

In all the experiments, the visual expression of the alterations caused by the three stress conditions (Foph, AF, and WD) was evaluated by monitoring the following symptoms: discoloration of lower leaf edges, hyponasty of lower leaves, turgor loss, leaf curling, wilting, and defoliation.

In experiment 1, symptoms of turgor loss and discoloration associated with Foph infection were assessed in the cape gooseberry plants. Based on such symptoms, the number of diseased plants (*Pd*) was determined considering the total number of plants (*Pt*). With the obtained data, disease incidence was calculated using Equation 1 (Madden et al., 2007).

(1) $$\begin{array}{l} I={\left(Pd/Pt\right)}^{*}100 \end{array}$$

In experiment 3, disease intensity in each treatment was estimated by calculating the area under the disease progress curve (AUDPC) by the trapezoidal integration method according to Jones (1998) using Equation 2,

(2) $$\begin{array}{l} AUDPC=\overset{\left(n-1\right)}{\underset{\left(i=1\right)}{\sum }}\frac{\left(y_{i}+y_{y+1}\right)}{2}\times \left(t_{i+1}-t_{i}\right) \end{array}$$

where n is the number of evaluations, *y*_*i*_ and *y*_*i*+1_ are the values of the severity scale that were observed at each time of evaluation, and *(ti* + *1 – ti*) is the time interval between evaluations.

Vascular browning was assessed at 0, 3, 6, 9, 12, and 15 DAT in cross stem sections taken from the base of the plants in each treatment. The percentage of browning of vascular bundles was estimated using a five-grade scale where 1 = no vascular browning; 2 = 1–25% vascular browning; 3 = 26–50% vascular browning; 4 = 51–75% vascular browning; 5 ≥ 75% vascular browning (Mandal et al., 2008; Chávez-Arias et al., 2020).

### Physiological Variables: Stomatal Conductance, Relative Water Content, Leaf Temperature, Plant Growth, and Biochemical Analysis

Leaf stomatal conductance (*g*_*s*_) was measured with a porometer (SC-1, Decagon Devices Inc., Pullman, WA, United States) in all the three experiments. A continuous excitation fluorometer (Handy PEA, Hansatech, Kings Lynn, United Kingdom) was used to determine the ratio of variable to maximum chlorophyll fluorescence (F_v_/F_m_). The F_v_/F_m_ ratio was obtained by keeping the leaf under evaluation in the dark for 15 min before each assessment (Maxwell and Johnson, 2000). All variables were measured on the third leaf in three plants per treatment in all the experiments. Finally, the F_v_/F_m_ readings registered in the second experiment were used to estimate the decrease in the maximum quantum efficiency of PSII (DQE). DQE was calculated using Equation 3 (Cháves-Gómez et al., 2020):

(3) $$\begin{array}{l} \text{DQE }=\left[{\text{F}}_{\text{v}}/{\text{F}}_{\text{m}}\text{ CP}-\left({\text{F}}_{\text{v}}/{\text{F}}_{\text{m}}\text{ FA}\right)/\left({\text{F}}_{\text{v}}/{\text{F}}_{\text{m}}\text{ CP}\right)\right]\;\times 100 \end{array}$$

where *CP* represents control plants (without exposure to *FA*) and *FA* represents plants exposed to FA concentrations.

The number of leaves and plant height were registered as plant growth variables. The plant height was recorded from the root neck to the apical meristem of the stem with a vernier caliper. All plant expanded leaves were considered to determine the number of leaves.

Leaf temperature and relative water content (RWC) were only measured in the third experiment (WD experiment). Leaf temperature was measured by an infrared thermometer (Cole-Parmer 800, Vernon Hills, IL, United States) on the same leaves used in *g*_*s*_ and F_v_/F_m_ ratio. The RWC was calculated by cutting a portion of tissue from the third leaf with a hole punch. The plant material was weighed (fresh weight, Fw) and brought to a humid chamber in a refrigerator at 4°C for 18 h; then, it was weighed again (turgid weight, Tw). Subsequently, the sample was taken to an oven at 65°C until reaching constant mass (dry weight, Dw). With the data obtained, the RWC was calculated using Equation 4 (Smart and Bingham, 1974).

(4) $$\begin{array}{l} RWC={\left(Fw-Dw/Tw-Dw\right)}^{*}100 \end{array}$$

The variables lipid peroxidation (malondialdehyde, MDA) and proline content were determined in the second and third experiments. The MDA content was estimated using the methods described by Hodges et al. (1999), and the method described by Bates et al. (1973) was used for proline production. For the determination of proline, 0.5 g of leaves used to assess physiological variables was macerated in liquid nitrogen and then added to 10 ml of 3% sulfosalicylic acid. The solution was filtered through filter paper; then, 2 ml of the filtrate was added to 2 ml of acidic ninhydrin plus 2 ml of acetic acid in a test tube for 1 h at 100°C, and the reaction was stopped with ice. Subsequently, toluene was added, and the solution was centrifuged at 3,000 g for 20 s. The supernatant was collected, and the absorbance was measured at 520 nm with a spectrophotometer (Spectronic BioMate 3 UV-vis, Thermo, Madison, WI, United States) using toluene as blank. Proline concentration was determined from the calibration curve. The thiobarbituric acid (TBA) method described by Hodges et al. (1999) was used to estimate membrane lipid peroxidation (MDA). Approximately 0.3 g of leaf tissue was homogenized in liquid nitrogen. Then, the samples were centrifuged at 5,000 rpm, and later the absorbances were measured at 440, 532, and 600 nm with the spectrophotometer. Finally, an extinction coefficient (157 M ml^−1^) was used to determine MDA concentrations.

### Data Analysis

Analyses of variance were performed on the data to compare the effect of the different treatments on the three experiments. A completely randomized design was utilized in the first experiment. The second and third experiments were analyzed with a factorial design. All percentage values were transformed using the arcsine function before analysis. If a significant F-test was observed, mean separation between treatments was obtained by the Tukey's test. Data were analyzed using the free software R 4.0.4 (R Core Team, 2021) (PBC, Boston, MA, United States).

## Results

### Experiment 1. Cape Gooseberry Plant Responses to *Fusarium oxysporum* f. sp. *physali* Inoculation

**Figure 1:**
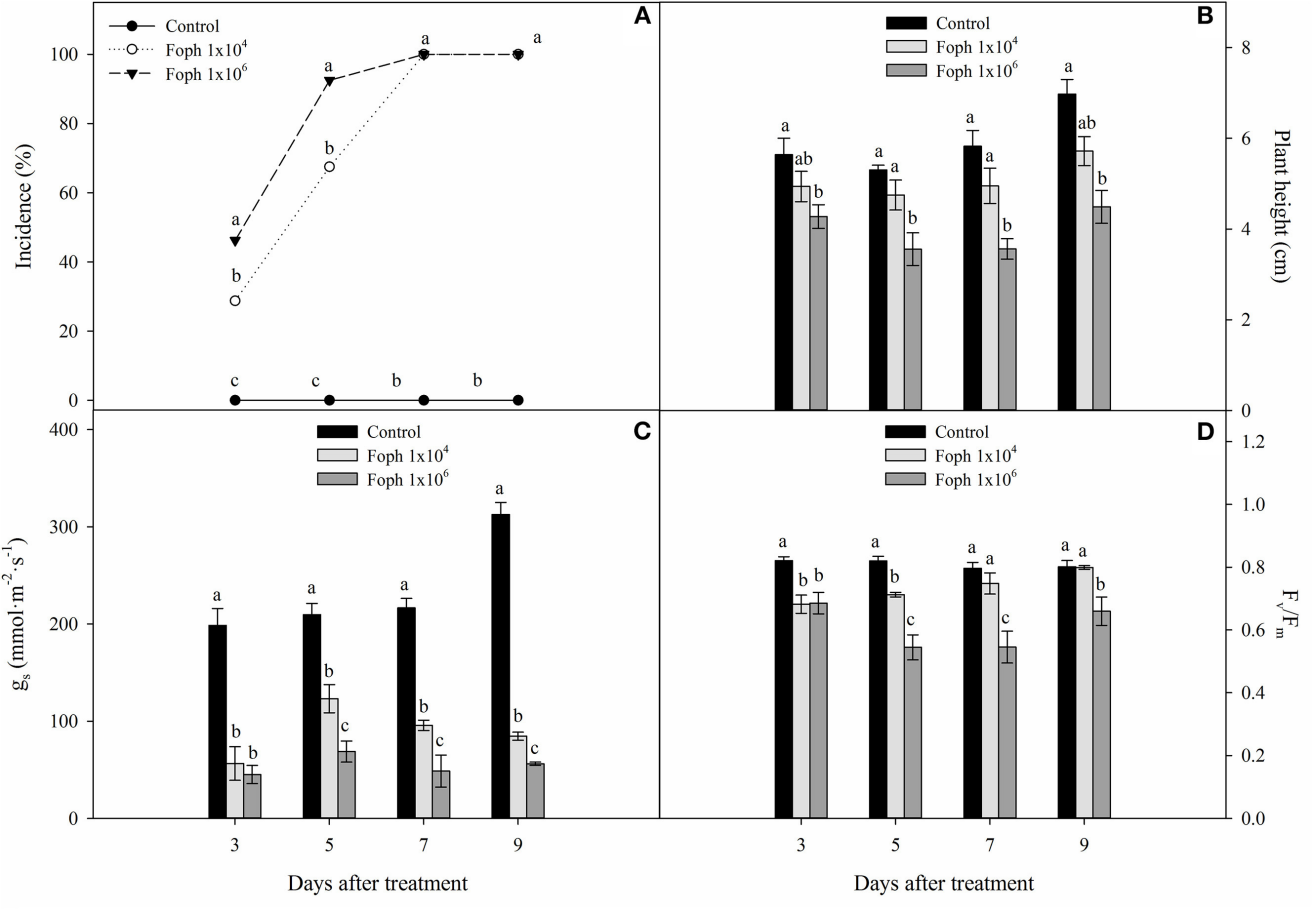
Development of vascular wilt caused by *Fusarium oxysporum* f. sp. *physali* (Foph) in cape gooseberry plant ecotype Colombia and variables of growth and plant physiology in a hydroponic system. **(A)** Incidence of disease under two inoculum densities, **(B)** plant height, **(C)** stomatal conductance (*g*_*s*_), and **(D)** quantum yield of photosystem II (F_v_/F_m_). Each bar or point chart represents the mean of three values ± standard error (*n* = 3). Bars or points followed by different letters indicate statistically significant differences according to the Tukey test (*P* ≤ 0.05).

Typical symptoms of the disease such as leaf turgor loss, wilting, drying, and defoliation were observed in the Foph-inoculated plants. The incidence of the disease caused by Foph was progressive, and the cape gooseberry plants showed symptoms as early as 3 DAT. Regarding the evaluated inoculum densities, the greatest disease values were obtained with an inoculum density of 1 × 10^6^ conidia ml^−1^ compared with 1 × 10^4^ conidia ml^−1^ at 3 and 5 DAT. However, the incidence of vascular wilt reached 100% for both treatments at 7 DAT (Figure 1A). The presence of the pathogen in inoculated plants was confirmed at 9 DAT by isolates obtained on PDA medium. Neither Fusarium wilt symptoms nor isolates of the pathogen on the PDA medium were observed for control plants. Regarding plant height, seedlings inoculated at 1 × 10^4^ conidia ml^−1^ did not show differences compared with control plants throughout experiment 1. However, plants exposed to a high inoculum density (1 × 10^6^ conidia ml^−1^) showed lower values in contrast to seedlings inoculated with 1 × 10^4^ conidia ml^−1^ and control plants at the end of experiment 1 (control: 6.9 cm, 1 × 10^4^ conidia ml^−1^: 5.7 cm, 1 × 10^6^ conidia ml^−1^: 4.4 cm) (Figure 1B). Also, Foph inoculation with both inoculum densities reduced stomatal conductance (*g*_*s*_) compared with the control plants throughout the experiment, observing that the seedlings inoculated with 1 × 10^6^ conidia ml^−1^ registered the lowest *g*_*s*_ at the end of experiment 1 (control: 312.8. mmol m^−2^ s^−1^, 1 × 10^4^ conidia ml^−1^: 83.3 mmol m^−2^ s^−1^, 1 × 10^6^ conidia ml^−1^: 56.1 mmol m^−2^ s^−1^) (Figure 1C). Finally, the quantum yield of photosystem II (F_v_/F_m_ ratio) of inoculated plants was generally lower in contrast to the control at all sampling points. At 9 DAT, the concentration of 1 × 10^6^ conidia ml^−1^ caused a higher drop in this ratio compared with the other treatments (control: 0.8, 1 × 10^4^ conidia ml^−1^: 0.79, 1 × 10^6^ conidia ml^−1^: 0.66) (Figure 1D).

### Experiment 2. Response of Cape Gooseberry Plants to Different Fusaric Acid Concentrations and Exposure Periods

**Figure 2:**
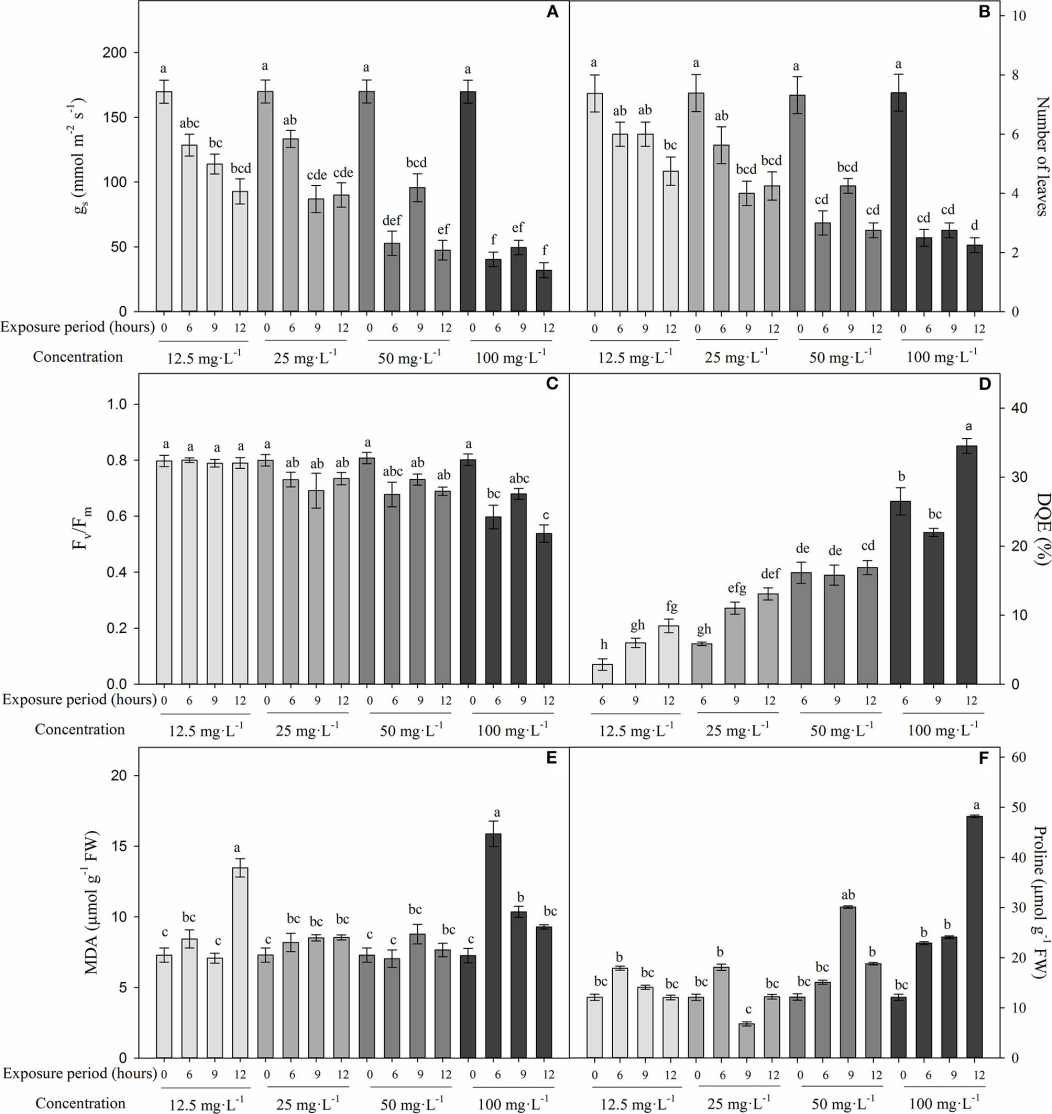
Effect of four concentrations (12.5, 25, 50, and 100 mg L^−1^) of fusaric acid (FA) and three exposure periods (6, 9, and 12 h) on the development of cape gooseberry plant ecotype Colombia in a hydroponic system 9 days after treatment. **(A)** Stomatal conductance (*g*_*s*_), **(B)** number of leaves **(C)** quantum yield of photosystem II (F_v_/F_m_), **(D)** maximum efficiency of PSII (DQE), **(E)** malondialdehyde (MDA) production, and **(F)** proline content. Bars related to 0 h exposure period correspond to control treatment for each evaluated FA concentration. Chart bars represent the mean of three values ± standard error (*n* = 3). Bars followed by different letters indicate statistically significant differences according to the Tukey test (*P* ≤ 0.05).

Figure 2 shows that differences were found between FA concentrations and exposure periods on stomatal conductance (*g*_*s*_) (*P* = 0), number of leaves (*P* = 0.002), F_v_/F_m_ ratio (*P* = 0.004), MDA (*P* = 0), and proline contents (*P* = 0) during experiment 2. In general, *g*_*s*_ and number of leaves significantly diminished when the FA concentration and time of exposure increased. The variables *g*_*s*_ (~40.6 mmol m^−2^ s^−1^) and number of leaves (~2.5) were lower in cape gooseberry plants subjected to different periods (6, 9, and 12 h) and at a concentration of 100 mg L^−1^ of FA. At a concentration of 50 mg L^−1^ of FA, the plants showed intermediate values of *g*_*s*_ and number of leaves with exposure periods of 6 and 12 h, respectively (average values of 50.1 mmol m^−2^ s^−1^ for *g*_*s*_ and 2.9 for number of leaves). Finally, plants grown with different FA concentrations (12.5, 25, 50, and 100 mg L^−1^) and recently established in the nutrient solution (without exposure period) showed the highest *g*_*s*_ (169.9 mmol m^−2^ s^−1^) and number of leaves (7.4) (Figures 2A,B).

The F_v_/F_m_ ratio also decreased significantly in plants grown at a concentration of 100 mg L^−1^ with exposure periods of 6 (0.6) and 9 (0.54) h compared with plants without any FA exposure time (with readings around 0.8) (Figure 2C). The decrease in the maximum efficiency of PSII (DQE) corroborated that high FA concentrations caused a greater affectation on PSII efficiency, since this group of plants showed percentage values between 25 and 35%. DQE values in the range between 25 and 45% indicate that the plants showed moderate damage (Figure 2D).

**Figure 3:**
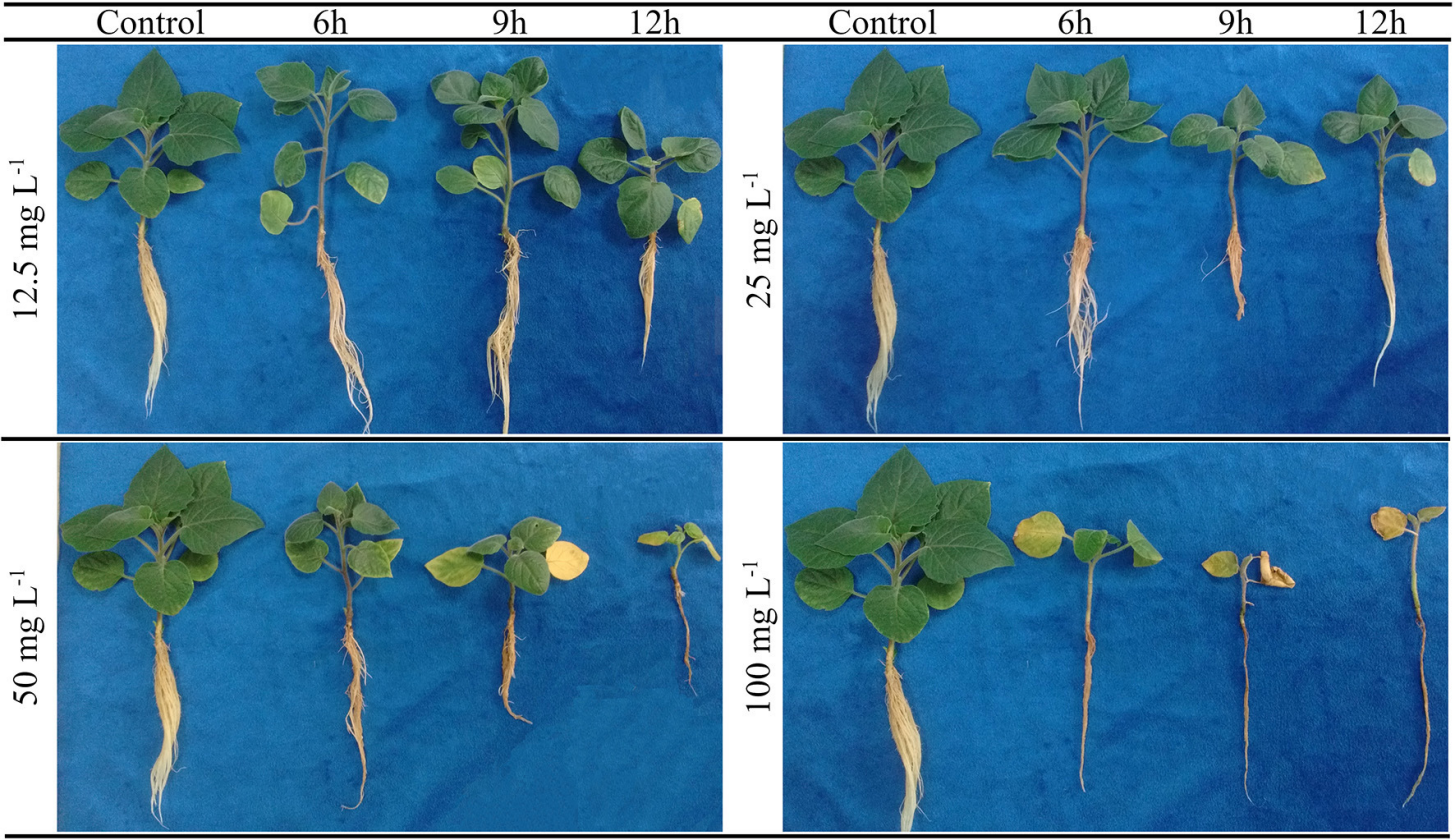
Effect of four concentrations (12.5, 25, 50, and 100 mg L^−1^) of fusaric acid and three exposure periods (6, 9, and 12 h) on the development of symptoms of cape gooseberry plant ecotype Colombia in a hydroponic system 9 days after treatment.

Low membrane lipid peroxidation (MDA) and proline synthesis were observed when the cape gooseberry plants were subjected to different FA concentrations. An increase in the MDA content was observed mainly in plants grown with FA concentrations of 12.5 and 100 mg L^−1^ and exposure periods of 6 and 12 h (13.4 and 10.3 μmol g^−1^ FW), respectively (Figure 2E). On the other hand, proline content was higher in cape gooseberry plants exposed to a FA concentration of 100 mg L^−1^ for 12 h (48.8 μmol g^−1^ FW) (Figure 2F). Finally, Figure 3 shows the effects of the FA concentrations and exposure periods on root and shoot growth of the cape gooseberry seedlings.

### Alteration in Plants Under Conditions of Water Deficit, Fusaric Acid, and *Fusarium oxysporum* f. sp. *physali*

**Figure 4:**
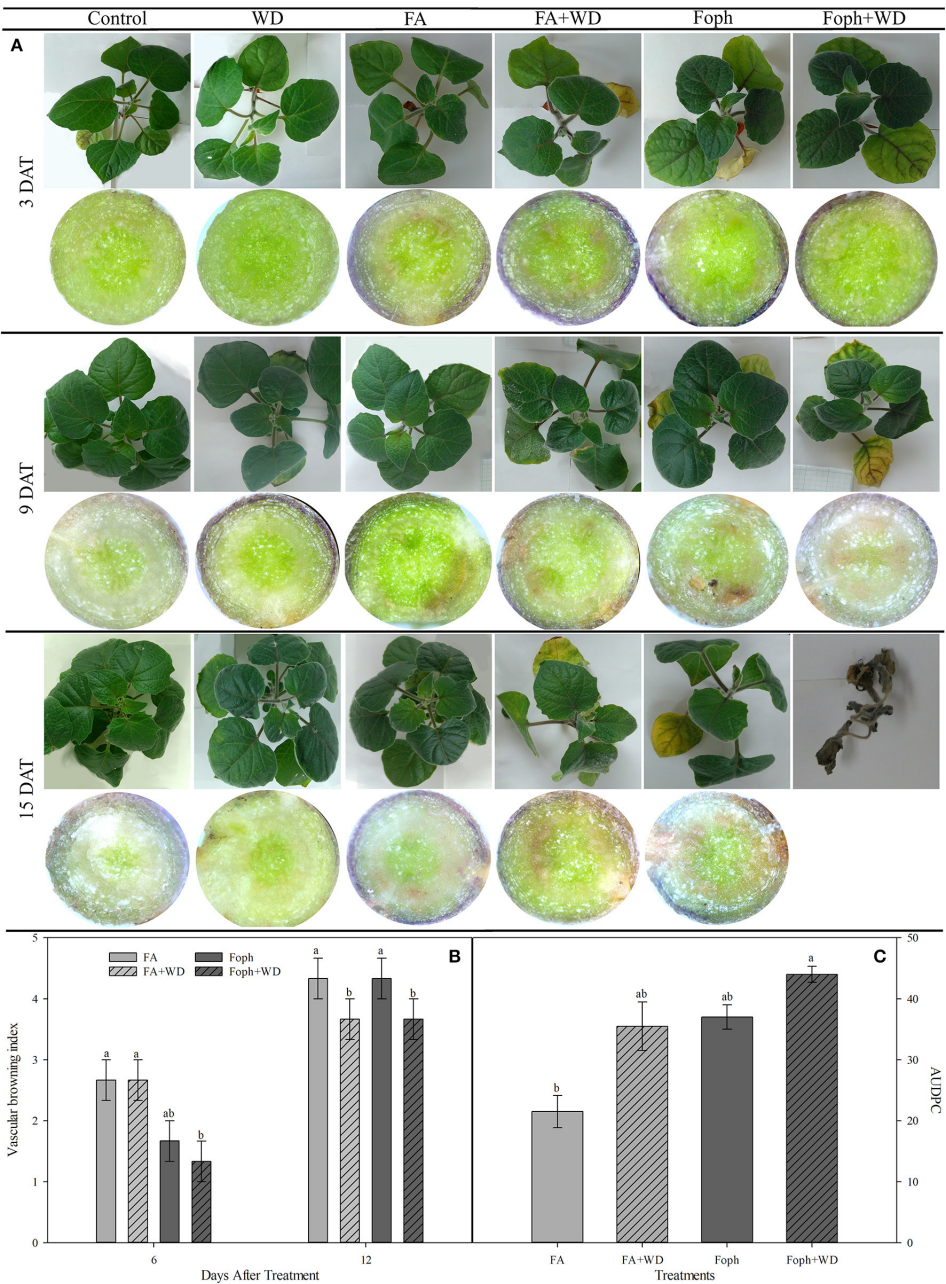
Effect of fusaric acid (FA), *F. oxysporum* f. sp. *physali* (Foph) and water deficit (WD) on cape gooseberry ecotype Colombia in a hydroponic system for 15 days. **(A)** Plant symptoms and their vascular bundles, **(B)** vascular browning index, and **(C)** area under disease progress curve (AUDPC). In **(B)** and **(C)**, neither vascular browning nor disease symptoms were observed in control plants; therefore, their values for each evaluated variable were 0. DAT, days after treatment. Chart bars represent the mean of four values ± standard error (*n* = 3). Bars followed by different letters indicate statistically significant differences according to the Tukey test (*P* ≤ 0.05).

Plants mainly subjected to FA + WD, Foph + WD, and WD started to show slight symptoms of leaf chlorosis, hyponasty, and dehydration between 3 and 9 DAT. Then, the evolution of symptoms showed that almost all treatments (FA, FA + WD, Foph, and Foph + WD) displayed strong leaf chlorosis and plant necrosis at the end of the experiment (15 DAT). Higher vascular browning was also observed in plants mainly exposed to FA and Foph (with or without WD) (Figure 4A). These symptoms in stems are related to the vascular browning index. Plants treated with FA or inoculated with Foph showed the highest values of this index (average from four values) at 12 DAT (Figure 4B). At the end of this experiment (15 DAT), plants exposed to Foph + WD were the only ones that died from those displaying wilt symptoms. The presence of the pathogen in inoculated plants was confirmed at 9 DAT by isolates obtained on the PDA medium. Neither Fusarium wilt symptoms nor isolates of the pathogen were observed on the PDA medium for control plants. The AUDPC analysis corroborated that this treatment (Foph + WD) showed the highest values of the disease (44), whereas the lowest values were observed in plants exposed to FA with a mean value of 21.5 (Figure 4C).

**Figure 5:**
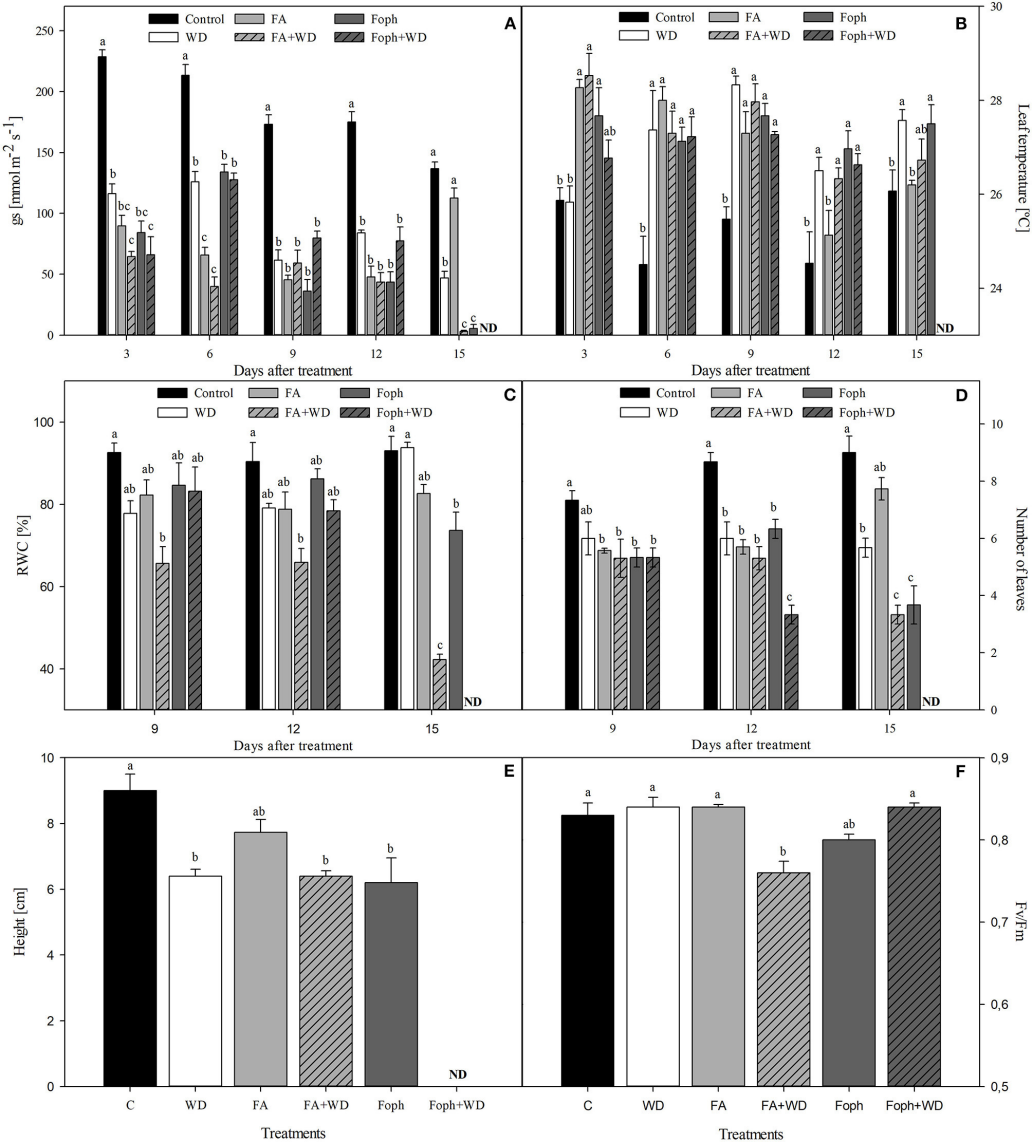
Effect of water deficit (WD), exposure to fusaric acid (FA), and inoculation with *F. oxysporum* f. sp. *physali* (Foph) on physiological variables of cape gooseberry plant ecotype Colombia in a hydroponic system. **(A)** Stomatal conductance, **(B)** leaf temperature, **(C)** leaf relative water content (RWC), **(D)** number of leaves, **(E)** plant height at 15 DAT, and **(F)** quantum yield of photosystem II (Fv/Fm) at 6 DAT. Bars represent the standard error. Different letters show significant differences (*P* ≤ 0.0*5*). Chart bars represent the mean of four values ± standard error (*n* = 3). Bars followed by different letters indicate statistically significant differences according to the Tukey test (*P* ≤ 0.05). ND, not determined.

Stomatal conductance (*g*_*s*_) was reduced in plants under WD, FA, FA + WD, Foph, and Foph + WD treatments compared with control plants at 3 and 9 DAT. It was also found that *g*_*s*_ decreased in plants under conditions of combined stress [FA + WD (5.5 mmol m^−2^ s^−1^)] or Foph inoculation (3.1 mmol m^−2^ s^−1^) in contrast to the control plants (136.8 mmol m^−2^ s^−1^) at the end of experiment 3 (Figure 5A). The variable foliar temperature confirmed what was found for *g*_*s*_, since plants treated with WD, FA, FA + WD, Foph, or Foph + WD showed higher values of foliar temperature (26.5–28.5°C) compared with the control (24.5–25.5°C) at different sampling points (Figure 5B).

The RWC was lower mainly in plants under FA + WD in experiment 3 at 9 (66.6%), 12 (65.86%), and 15 (45.2%) DAT, respectively (Figure 5C). Regarding plant growth parameters, the combined stresses (FA + WD or Foph + WD) or only Foph inoculation caused the most negative effects on the number of leaves and plant height at the end of the experiment (Figures 5D,E). In terms of F_v_/F_m_, significant differences (*P* = 0) were found among the treatments, where plants under FA + WD showed the lowest mean value of 0.76 compared with the other evaluated treatments at 6 DAT (Figure 5F).

**Figure 6:**
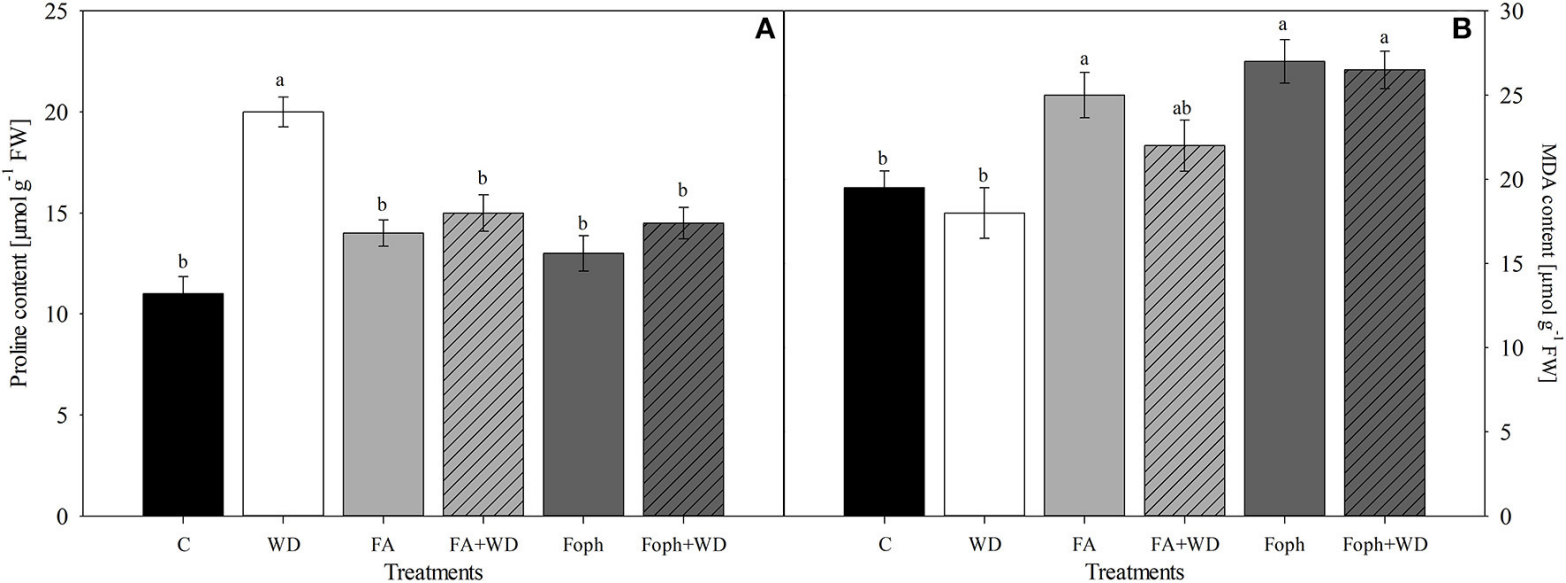
Effect of water deficit (WD), exposure to fusaric acid (FA) and *F. oxysporum* f. sp. *physali* (Foph) inoculation and combination of WD and FA and WD and Foph on proline and malondialdehyde contents in cape gooseberry plant ecotype Colombia under hydroponic system conditions at 12 DAT. **(A)** Proline and **(B)** malondialdehyde (MDA) contents. Chart bars represent the mean of four values ± standard error (*n* = 3). Bars followed by different letters indicate statistically significant differences according to the Tukey test (*P* ≤ 0.05).

Plants under WD showed the highest proline content compared with the other treatments (Figure 6A). On the other hand, MDA content also showed significant differences (*P* = 0.014) for this variable. Plants grown under FA, Foph, or Foph + WD treatments produced 10% more MDA than the control plants (Figure 6B).

## Discussion

Cape gooseberry crops are severely affected by *Fusarium* wilt caused by Foph in most production areas in Colombia. Recent studies have contributed to the understanding of cape gooseberry plant responses to waterlogging and Foph infection (Chaves-Gómez et al., 2019; Chávez-Arias et al., 2020). Nevertheless, plant responses to drought and Foph exposure had not been evaluated before. The results of the three experiments conducted helped to elucidate the interaction between Foph and cape gooseberry under normal or stress conditions (single or combined). Visual and physiological plant reactions to these stresses were characterized. Additionally, this study allowed to expand the knowledge of the use of FA as a virulence factor to determine possible physiological and biochemical plant responses as acclimatization mechanisms under combined stress (Foph and water deficit). Predicting the effects of water stress under Foph infection in a climate change scenario is necessary to develop crop management strategies to mitigate the negative impact of abiotic and biotic stress conditions.

Plant wilting and defoliation were the main responses to both inoculum densities in cape gooseberry (experiment 1). These plant symptoms were the result of pathogen infection and its influence on water uptake and transport from roots to shoots (Husaini et al., 2018; Chaves-Gómez et al., 2019; Zumaquero et al., 2019; Chávez-Arias et al., 2020). An inoculum concentration of 1 × 10^6^ conidia ml^−1^ caused the highest plant wilting and defoliation. Studies carried out by Martyn and McLaughlin (1983) and Moreno-Velandia et al. (2019) found that an inoculum level of 1 × 10^6^ conidia ml^−1^ caused similar plant symptoms to those described above. These authors also consider this inoculum density to be suitable for ranking susceptible or tolerant genotypes or evaluating agronomic strategies to manage Fo.

Fusaric Acid caused leaf chlorosis and loss of turgor, stem necrosis, root damage, and a decrease in plant biomass (experiment 2). Plants exposed to this toxin may have shown effects similar to those of Fo infection, such as necrosis, chlorosis, and curling of upper leaves (Bouizgarne et al., 2006; Dong et al., 2012, 2014; Wang et al., 2015). As a consequence, according to the symptoms observed, the results confirmed that healthy cape gooseberry plants developed wilt disease symptoms when exposed to the mycotoxin FA. This finding fulfills one of the two conditions stated by Aducci et al. (1997) to consider a toxin a disease determinant. In Foph, the presence of SIX (secreted in xylem) homologous and putative effectors clarified the interaction between Foph and cape gooseberry (Simbaqueba et al., 2021). In this study, mechanisms that contribute to Foph pathogenicity and virulence were elucidated. Brown et al. (2012, 2015) reported FA biosynthesis to be mediated by *FUB* genes *(FUB1–FUB12*). Nine of these genes are involved in FA biosynthesis, but they have not been studied in Foph. As the biosynthesis mechanisms of FA in Foph were not determined in this research, further studies are needed to deepen in the role of this phytotoxic secondary metabolite in vascular wilt in cape gooseberry plants. As FA is (i) reported in *formae specials* of Fo such as *F. oxysporum* f. sp. *cubense* (Liu et al., 2020), *F. oxysporum* f. sp. *lycopersici* (Yun et al., 2019) *F. oxysporum* f. sp. *lilii* (Curir et al., 2000), *F. oxysporum* f. sp. *albedinis* (Bouizgarne et al., 2004), *F. oxysporum* f. sp. *vasinfectum* (Stipanovic et al., 2011), *F. oxysporum* f. sp. *benincasae* (Xie et al., 2011), *F. oxysporum* f. sp. *medicagenis* (Geraldo et al., 2010), *F. oxysporum* f. sp. *gladioli* (Nosir et al., 2011), *F. oxysporum* f. sp. *niveum* (Chunli et al., 2000), *F. oxysporum* f. sp. *cucumerinum* (Wang et al., 2015), *F. oxysporum* f. sp. *physali* [strain MAP5 used in this study, according to Izquierdo-García and Moreno-Velandia, (2018) (personal communication)] and many *Fusarium* species, and (ii) is a non-specific toxin (Bacon et al., 1996; Niehaus et al., 2014), the findings may be considered as the first approach to the study of the role of FA in cape gooseberry wilt caused by Foph.

Singh and Upadhyay (2014) also mentioned that the disease symptoms caused by FA depend on concentration and exposure time. Results from experiment 2 showed that FA caused a phytotoxic effect and physiological affectations (low stomatal conductance and high MDA content) after an exposure period of 6 h at all evaluated concentrations. Singh et al. (2017) observed differences in stomatal conductance and MDA content after an exposure of 4 h in FA-treated tomato plants. These authors also stated that the *Fusarium* toxin damages the photosynthetic apparatus because of the development of oxidative stress. Lipid peroxidation has been used as a marker of oxidative stress indicating damage to the plasma membranes of cells by MDA production (AbdElgawad et al., 2016; Gonçalves et al., 2017). Therefore, the findings show that exposure to the treatment with FA led to an increase in lipid peroxidation (expressed as a higher MDA content). These observations match the plant alterations observed in this study, with the most severe symptoms being associated with the increase in FA concentration (Boari et al., 2003).

Chlorophyll fluorescence parameters in terms of F_v_/F_m_ ratio can be used as a fast and non-destructive method that allows the assessment of plant tolerance estimation or acclimatization to biotic stress conditions (Chávez-Arias et al., 2019). F_v_/F_m_ ratio reflects the complete PSII functioning, and a decrease in its values is related to the impairment of PSII under stress conditions (Cháves-Gómez et al., 2020). In this study, F_v_/F_m_ was severely reduced by Foph inoculation (experiment 1) and FA exposure (experiment 2). These results are in accordance with the findings of Dong et al. (2012). These authors reported a significant reduction in F_v_/F_m_ ratio in banana plants with FA exposure. Similarly, Cháves-Gómez et al. (2020) reported a reduction in F_v_/F_m_ values after Foph inoculation in cape gooseberry plants. A decrease in the F_v_/F_m_ ratio due to Foph inoculation and FA exposure suggests damage at the chloroplast level caused by oxidative stress. This oxidative stress may promote a down-regulation of the electron transport process and photodamage at the PSII reaction centers. Additionally, CO_2_ assimilation (reduction of photosynthesis activity) in plant leaves may be observed as a secondary effect of this stress (Nogués et al., 2002; Pshibytko et al., 2006; Dong et al., 2012).

The development of vascular wilt caused by Fo in plants blocks vascular bundles, inducing their necrosis (Husaini et al., 2018; Chaves-Gómez et al., 2019; Zumaquero et al., 2019; Chávez-Arias et al., 2020). This was also observed in cape gooseberry plants inoculated with Foph. These results appeared simultaneously with visual symptoms of the disease and vascular bundle colonization by the pathogen, confirmed by Foph isolation from diseased plant tissues on PDA medium. Browning of vascular bundles was also observed in plants exposed to FA as reported by several authors (Bacon et al., 1996; Bouizgarne et al., 2006; Dong et al., 2012; Singh et al., 2017). Necrosis or browning are caused by cell death induced by FA, since this phytotoxin causes membrane permeability changes, dysfunctions of mitochondrial activity, and inhibition of respiration (Singh et al., 2017).

The severity of damage was higher in Foph-inoculated or FA-treated plants under WD conditions (experiment 3). WD causes a decrease in the water potential of the root growth medium, which reduces water availability for the plant (Farooq et al., 2009). This effect was reflected in low RWC levels for the FA + WD, Foph + WD, and WD treatments. Plant height and number of leaves in cape gooseberry seedlings were altered under both biotic conditions (toxin and pathogen) and worsened under WD conditions. These results are similar to those reported in melon plants inoculated with *F. oxysporum* f. sp. *melonis* under WD conditions (Jorge-Silva et al., 1989). This may suggest that WD conditions accelerate the appearance and intensity of symptoms in cape gooseberry plants. The results obtained in this study are consistent with research carried out in other plant species (Wu et al., 2008b). Additionally, physiological alterations (low stomatal conductance) have been reported in plant species under biotic stress (FA or FO) (Dong et al., 2012, 2014; Wang et al., 2012). However, WD conditions caused a higher stomatal closure in cape gooseberry plants exposed to biotic stresses 12 DAT. A higher decrease in stomatal conductance in plants under combined stress conditions may be due to the fact that the plant seeks to minimize water loss through transpiration and maintain cellular homeostasis (Farooq et al., 2009), which in turn generates an increase in leaf temperature. Finally, water stress also induces a higher MDA synthesis in plants exposed to biotic stress (FA- or Foph-treated plants). Ma et al. (2015) stated that drought could greatly favor the spread of *Fusarium* and observed that a high level of MDA was detected in all the plants under WD conditions and *Fusarium* presence.

**Figure 7:**
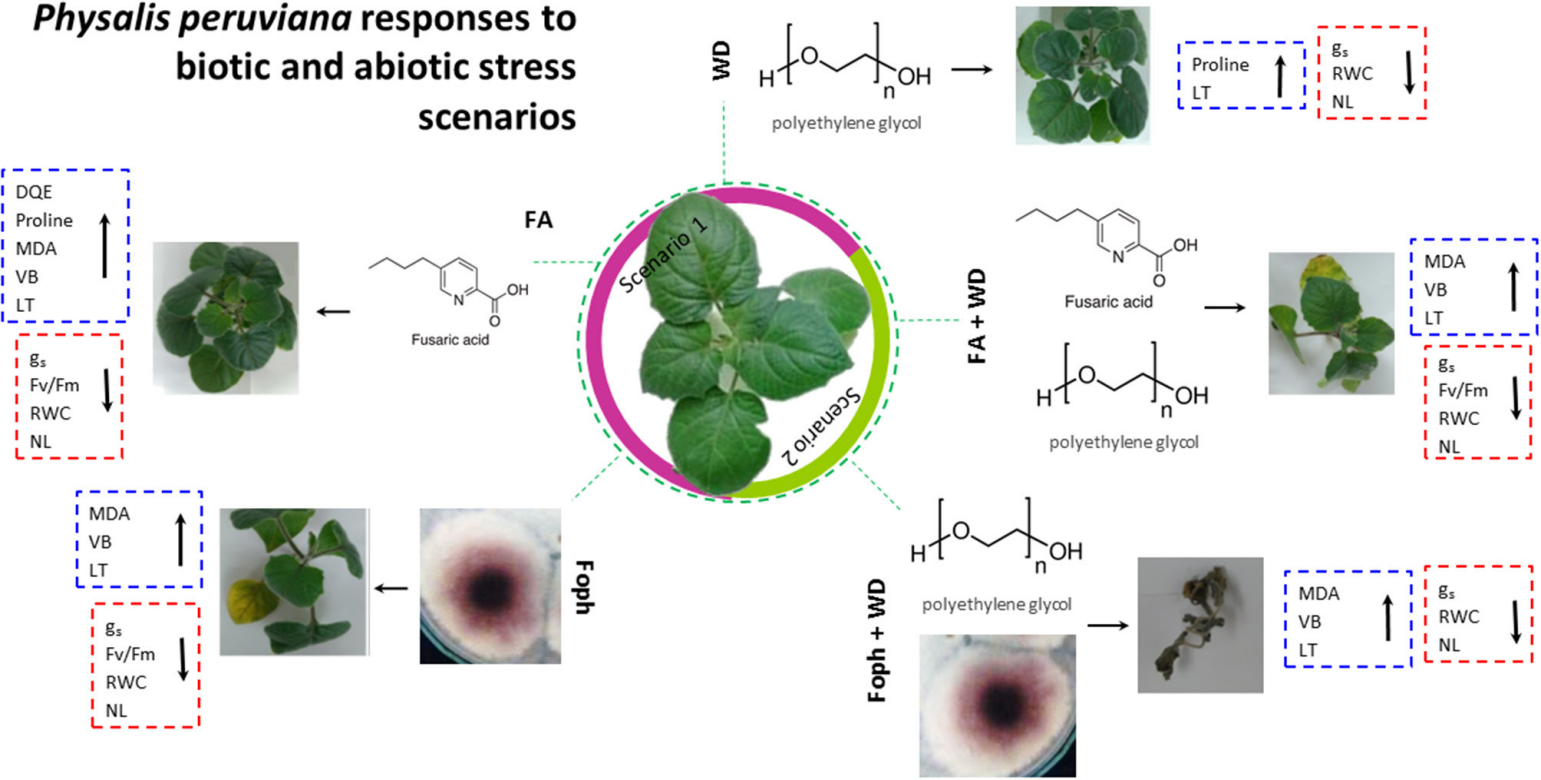
Schematic representation of *Physalis peruviana* ecotype Colombia responses to *Fusarium oxysporum* f. sp. *physali* (Foph) inoculation and water deficit (WD, polyethylene glycol) as potential biotic and abiotic plant stress scenarios. The model includes fusaric acid (FA) as one of the main *Fusarium* toxins involved in pathogenesis to visualize its role in plant disease development. The central cape gooseberry plant represents a healthy plant. Scenario 1 (purple area) is related to biotic stress caused by Foph and FA. Scenario 2 (green area) includes abiotic stress (WD) and its interaction with FA and Foph. Visual expression of the symptoms followed by plant physiological responses to each stress condition is shown. Physiological parameters that increase and decrease under each stress condition are clustered in red and blue dotted line boxes, respectively. From left to right: responses to (i) Foph Map5 strain, (ii) FA exposure, (iii) WD stress condition, (iv) WD stress condition under FA exposure, and (v) WD stress condition under Foph Map5 presence. VB, vascular browning; *g*_*s*_, stomatal conductance; F_v_/F_m_; quantum yield of photosystem II; DQE, maximum quantum efficiency of PSII; MDA, malondialdehyde; LT, leaf temperature; NL, number of leaves.

The responses of *P. peruviana* “Colombia” plants to two biotic and abiotic stresses (experiments 1, 2, and 3) are summarized in Figure 7. The results show common trends of the physiological responses of cape gooseberry plants to five stress conditions. Under stress caused by a single factor (Foph, FA, or WD) as scenario 1 (purple area), plant responses were similar to those detected when *P. peruviana* was exposed to combined stress conditions (Foph + DW, FA + DW). In both cases (single or combined exposure), *g*_*s*_, F_v_/F_m_, RWC, and NL decreased as plants remained under the exposure. In contrast, DQE, VB, LT, and MDA showed an increasing trend regardless of the type of stress. From the macroscopic level, Foph, FA, and WD caused high, medium, and low negative impacts, respectively, on cape gooseberry plants. In this scenario, the strongest negative effect was caused by Foph. This result shows that other virulence factors may be involved in vascular wilt development, although FA plays an important role in pathogenesis. On the other hand, a marked negative effect was observed on plant physiology responses under combined stress conditions in scenario 2 (green area in Figure 7). The results obtained revealed that WD triggers a stronger negative effect on cape gooseberry plants under Foph or FA exposure, with a lower impact of FA + WD compared with Foph + WD.

In this study, WD promoted the early appearance of symptoms and increased the negative effect on the physiology of cape gooseberry plants exposed to FA or inoculated with Foph. The findings are a contribution to the knowledge of the cape gooseberry-Foph pathosystem, and the effect of biotic and abiotic stress factors and their combination on the physiological response of plants of this species. Additionally, the results derived from this study bring out the potential effect of the two main abiotic and biotic stresses that impair growth and development in cape gooseberry plants. Among these stresses, Foph may be considered the main threat to this crop and, combined with WD, could become a limiting factor for crop sustainability. Therefore, crop management strategies should be driven to avoid the concurrent risk of these conditions or to mitigate their negative effect on plant physiology.

## Data Availability Statement

The raw data supporting the conclusions of this article will be made available by the authors, without undue reservation, to any qualified researcher.

## Author Contributions

LM-V, CC-A, HR-D, and SG-C: conceptualization and writing—review and editing. LM-V, WV-R, and AC-T: investigation and writing of the original draft. LM-V, CC-A, HR-D, and SG-C: validation. LM-V and CC-A: formal analysis and data curation. JR-G: critical reading and editing of the original draft. HR-D and SG-C: resources, supervision, project administration, and funding acquisition. All authors contributed to the article and approved the submitted version.

## Conflict of Interest

The authors declare that the research was conducted in the absence of any commercial or financial relationships that could be construed as a potential conflict of interest.

## Publisher's Note

All claims expressed in this article are solely those of the authors and do not necessarily represent those of their affiliated organizations, or those of the publisher, the editors and the reviewers. Any product that may be evaluated in this article, or claim that may be made by its manufacturer, is not guaranteed or endorsed by the publisher.
